# Visual Localization Based on Torus-like Surfaces

**DOI:** 10.3390/s24227349

**Published:** 2024-11-18

**Authors:** Xuandong Liu, Lihong Luo, Bingren Shen

**Affiliations:** 1 School of Computer Science and Technology, Guangdong University of Technology, Guangzhou 510006, China; 13828417894@163.com; 2 School of Digital Media, Guangdong University of Technology, Guangzhou 510006, China; shenbingren0@163.com

**Keywords:** visual localization, point correspondence, surface intersection, tracking method, augmented reality, indoor navigation

## Abstract

Previous visual localization started from point correspondences (PCs) to estimate poses. This article takes the camera position as the entry point and finds that the camera position solution set rotates around an axis connected by two observed 3D points to form a surface called a torus-like surface (TLS). The relevant parameters of TLS are calculated based on PCs and camera intrinsic parameters. In order to reduce the number of solutions, this article uses four PCs to construct three TLSs. By utilizing four PCs, the pose determination problem is reformulated as the task of finding the optimal intersection of three surfaces. By using the step-size adaptive tracking method, the candidate set of intersections can be quickly and accurately found. Combining the feature information of intersections on TLS and the camera intrinsic parameters, the optimal position is obtained. Based on this position, the rotation matrix can be determined. In the synthetic data experiments and the dataset experiments based on image localization, it is shown that the visual localization based on TLS is more accurate than current state-of-the-art methods, which provides a new entry angle and effective ideas for visual localization. Its accuracy and practicality are fully demonstrated in the application test of augmented reality indoor navigation.

## 1. Introduction

Mobile devices typically use inertial measurement units within the device for inertial localization navigation. However, inertial navigation algorithms have some problems, such as mismatch and inconsistent image features due to changes in camera position while the device is moving, which affects the accuracy and continuity of localization [1]. In addition, it may be disturbed by its own temperature, zero bias, vibration, and other factors to produce errors [2]. These errors will gradually accumulate during the moving process, affecting the device’s accurate cognition of its own position, which in turn leads to the deviation of the navigation path. Therefore, it is necessary to adopt correction algorithms. The most common correction method is image-based visual localization [3,4].

The dominant approach to image-based visual localization is to estimate the camera pose by querying the correspondences between the 2D features of the image and the known 3D points in the scene. These correspondences are likely to have mismatched information and thus need to be filtered with Random Sample Consensus (RANSAC) [5] or its derivatives [6] to find suitable correspondences. The most representative mainstream methods of this kind are P3P [7] and Infinitesimal Plane-based Pose Estimation (IPPE) [8]. P3P uses three PCs to estimate the camera position, but there is a dangerous cylinder problem [9] that makes the solution unstable, while IPPE requires four coplanar PCs to estimate the pose, which is accurate and stable but has more stringent requirements for coplanar 3D points.

In order to improve the accuracy and speed of absolute pose estimation, people have started to investigate feature information other than PCs. For example, local affine correspondences are obtained by combining 2D matching [10], or external pose information is introduced from the inertial measurement unit (IMU) [11], and the pose is estimated by single-point affine correspondences and an estimate of the surface normal at the 3D point [12].

In order to balance real-time estimation and accuracy, people are more likely to improve real-time estimation by reducing PCs while introducing other feature information for accuracy compensation. Therefore, we started to explore whether there is a method to maintain high real-time performance without reducing PCs but still solve the accurate position, and at the same time, the method does not impose harsh restrictions on the 3D points. To this end, we try to find the intrinsic connection between PCs and revisit the monocular imaging model, starting from the camera’s trajectory for the 3D points, and find that the camera trajectory forms a torus-like surface model at the two visible 3D points, and its view cone is always perpendicular to the tangent plane of the torus-like surface at the camera’s position, and by combining the camera’s internal references with the 3D points, we can easily obtain the 3D coordinates of the projection points that are projected onto the imaging surface. Following this, the rotation matrix is determined.

Compared with other methods, our idea is different. When the reference camera and the query camera observe the same four 3D points, the viewing angle will be adjusted following the different positions to ensure that the same four points are observed, three torus-like surfaces are constructed based on these four 3D points, and the optimal intersection of three torus-like surfaces will be used as the position solution for the camera, and then the rotation matrix is determined based on 2D-3D correspondences, camera intrinsics, and the information of the estimated position. This method estimates the position more accurately, and we demonstrate this through experiments on synthetic and real data.

Our contributions are as follows:

- We propose a novel method for solving the pose, which converts the problem of solving the pose with four PCs that are not colinear between three 3D points into a problem of intersecting surfaces to find intersections. We apply a tracking algorithm with an adaptive step size to determine surface intersections, which are stable and fast. Combining the intersection positions, the PCs, and camera intrinsics, the rotation matrix will be estimated;
- Experiments on synthetic data demonstrate the numerical stability of our approach for solving and analyzing its performance under various types of noise;
- Experiments on two benchmark datasets based on image localization show that our method can estimate more accurate camera pose than state-of-the-art methods in comparable time;
- Application testing of TLS visual localization applied to augmented reality in-door navigation to verify its utility and accuracy.

## 2. Related Work

Perspective-n-Points (PnP) is the problem of solving the camera pose by using n pairs of matching world and image coordinates when the object’s coordinates in the world coordinate system and its image coordinates are known. Previous research has obtained the following patterns after deduction: when n is 1 or 2, there are infinite solutions; when n is 3, there are at most four solutions; when n is 4 and four points in the world coordinate are coplanar, there is only one solution, etc. [13,14].

The PC-based PnP problem has a minimum n value of 3, which is also known as the P3P problem. The problem of estimating the absolute pose of a calibrated camera from three PCs has been considered for almost two centuries. Recent research has continued to improve the speed and stability of P3P solutions [15,16], including an updated method recently introduced [17].

In the past decade, many methods have been proposed to solve the PnP problem for n > 3. These methods can be roughly categorized into iterative and non-iterative.

Classical iterative methods formulate the PnP problem as an optimization objective function through the relationship between the 3D control points and the 2D projection points and then use an iterative optimization method such as Levenberg–Marquardt [18] or L-BFGS [19] to estimate the pose. For example, in [20,21], such methods are prone to fall into local minima without a suitable starting point, which can lead to degradation of accuracy.

A non-iterative approach looks for constraint information among the PCs to estimate the pose. Collins and Bartoli derived new constraints and minimum solutions for the absolute pose of the camera given four coplanar PCs by selecting an optimal point on a plane to compute a first-order approximation to the homography (an affine transformation) and then computed the camera’s pose based on the 2D point projections and the first-order approximation local transformations. Mills [22] describes a four-point solver for fundamental matrix estimation, while Barath [23] develops a five-point solver for estimating the fundamental matrix from oriented features.

Recently, constraint information other than PCs has been utilized to estimate the pose. Köser and Koch introduced a solution for absolute pose estimation from a single affine correspondence (AC) in orthogonal reference images [10], while Ventura et al. developed a generalized absolute pose solution from perspective references based on the knowledge of the surface normal. In order to reduce the minimum sample size of the absolute pose, [24] proposed introducing external attitude information from an inertial measurement unit (IMU) to solve the pose.

The above methods start from PCs or combine constraints other than PCs to estimate pose but do not start from the pose solution set to explore new constraints to estimate pose. We review the perspective geometric model, materialize the camera intrinsics and focal length as a view cone, explore what kind of motion trajectory the vertices of this view cone follow to determine the solution set of the camera position, and then find the exact pose solution.

## 3. Methods and Works

*P* and *Q* are two points in world coordinate, *OKLMN* is the view cone, point *O* is the camera position, and *p* and *q* are the projections of *P* and *Q* on imaging plane *KLMN*. Combining points *P*, *Q*, and *O*, we can define a plane *PQO*.

**Figure 1:**
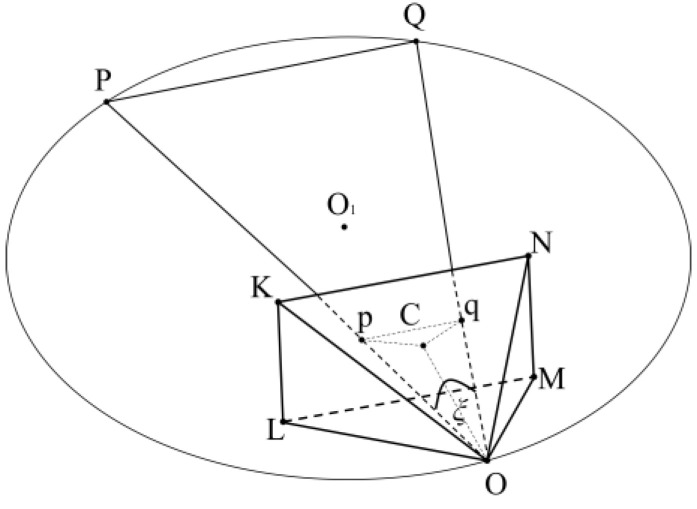
Trajectory of point *O* on the plane *OPQ*.

Under the condition that the view cone is guaranteed to be invariant (i.e., camera intrinsics are invariant), the trajectory of the solution set formed by the camera position *O* on the plane *OPQ* is shown in Figure 1. The trajectory is known to be a circular line, and the imaging angle *ξ* is unchanged when the view cone is moved on circular line in the plane *PQO.*

### 3.1. Solve for the Angle ξ

The horizontal field of view angle ∠*LOM*, the vertical field of view angle ∠*NOM,* and the physical focal length *OC* of the camera are obtained by calibrating the device camera, where *OC* is also the distance from the camera optical center *O* to the camera imaging plane *KLMN.*

The physical length of *KL* and *KN* can be obtained from the orthogonal equation:

(1) $$\begin{array}{l} KL=2\cdot \tan\left(\theta_{v}/2\right)\cdot OC\\ KN=2\cdot \tan\left(\theta_{h}/2\right)\cdot OC \end{array}$$

where *θ_h_* is ∠*LOM*, and *θ_v_* is ∠*NOM*.

Known image coordinates are projected on the imaging planes *p* and *q*; for the center image coordinates (*C*) of the imaging plane, the imaging plane height is represented by *h_img_*, width is represented by *w_img_*, and the physical length of the imaging plane is represented by *KL* and *KN*; the physical distance between *p*, *q*, and *C* is obtained by scaling *t*:

(2) $$\begin{array}{l} t=\frac{\sqrt{KL^{2}+KN^{2}}}{\sqrt{{h_{img}}^{2}+{w_{img}}^{2}}}\\ Cp=Cp_{img}\cdot t\\ Cq=Cq_{img}\cdot t\\ pq=pq_{img}\cdot t \end{array}$$

where *Cp_img_*, *Cq_img_*, and *pq_img_* are the Euclidean distances between image coordinates, and *Cp*, *Cq*, and *pq* are the physical lengths in the real world. Combining the physical focal length *OC* and Pythagorean theorem, the physical length of *Op* and *Oq* can be found. According to the cosine law, we can solve for *ξ* as follows:

(3) $$\xi =\arccos\left(\frac{Op^{2}+Oq^{2}-pq^{2}}{2\cdot Op\cdot Oq}\right)$$

### 3.2. Definition of Parametric Equation for Torus-like Surfaces

According to Figure 1, knowing the world coordinates of points *P* and *Q* and their angle *ξ* at point *O*, the radius *R* of the circular line, with *O*_1_ as the center and *PQ* as the chord, and the distance *h* from the center *O*_1_ to the chord *PQ* can be found:

(4) $$\begin{array}{c} R=\sqrt{\frac{PQ^{2}}{2\cdot (1-\cos(2\cdot \xi ))}}\\ h=R\cdot \cos(\xi ) \end{array}$$

**Figure 2:**
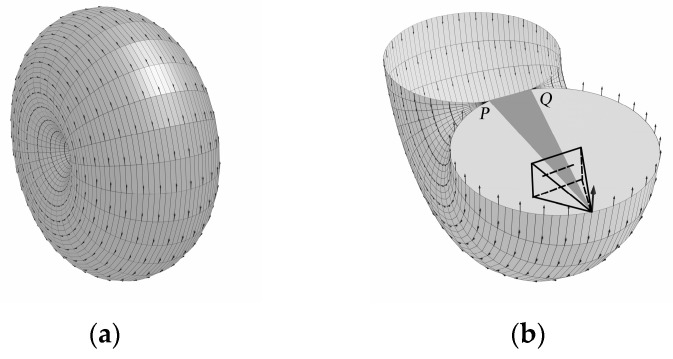
Torus-like surface field. (**a**) Complete; (**b**) lower half.

Figure 1 is just the trajectory of a point O on a plane, and there are countless such planes in world coordinate systems, each of which constructs a segment of arcs with PQ as the chord. These arcs will rotate with PQ as the axes to obtain a rotary body shaped like Figure 2a.

To facilitate the observation of the position of the view cone in relation to this rotated body, the lower half of it is given in Figure 2b. We will refer to the surface of this rotated body as the torus-like surface (TLS), where all points on the TLS are the possible solution set of camera position *O.*

It is easy to see from the generation process that a TLS at any position in the world coordinate system is obtained by rotating an arc around any axis. Taking the *PQ* axis as an example, its parametric equations are essentially the parametric equations of a spatial circumference with radius *h* determining the trajectory of the center of the circular arc and determining the size of the arc based on the center of the spatial circumference with radius *R.*

For the general equation for the spatial circumcircle with the midpoint of the *PQ* axis as the center (*x*_mid_, *y*_mid_, *z*_mid_), vector ***PQ*** as the normal vector (*A*_1_, *B*_1_, *C*_1_), and *h* as the radius, Equation (5) represents the trajectory obtained by rotating the center of the circle *O*_1_ around the ***PQ*** in Figure 1:

(5) $$\;\left\{\begin{array}{c} {\left(x_{O_{1}}-x_{mid}\right)}^{2}+{\left(y_{O_{1}}-y_{mid}\right)}^{2}+{\left(z_{O_{1}}-z_{mid}\right)}^{2}=h^{2}\\ A_{1}(x_{O_{1}}-x_{mid})+B_{1}(y_{O_{1}}-y_{mid})+C_{1}(z_{O_{1}}-z_{mid})=0 \end{array}\right.$$

To facilitate the solution, it needs to be converted into a parametric equation. Let *X* = *x_O_*_1_ − *x*_mid_, *Y* = *y_O_*_1_ − *y*_mid_, and *Z* = *z_O_*_1_ − *z*_mid_ for Equation (5). Combining the two equations, the *Z* variables and formulas are deformed to obtain Equation (6):

(6) $$\;\frac{\left({A_{1}}^{2}+{B_{1}}^{2}+{C_{1}}^{2}\right){C_{1}}^{2}}{{B_{1}}^{2}+{C_{1}}^{2}}X^{2}+(\frac{A_{1}B_{1}}{\sqrt{{B_{1}}^{2}+{C_{1}}^{2}}}X+\sqrt{{B_{1}}^{2}+{C_{1}}^{2}}Y)=h^{2}{C_{1}}^{2}$$

To convert (6) into a more easily computable parametric equation, let

(7) $$\;\left\{\begin{array}{c} X=h\sqrt{\frac{\left({B_{1}}^{2}+{C_{1}}^{2}\right)}{\left({A_{1}}^{2}+{B_{1}}^{2}+{C_{1}}^{2}\right)}}\cos u\\ \frac{A_{1}B_{1}}{\sqrt{\left({B_{1}}^{2}+{C_{1}}^{2}\right)}}X+\sqrt{\left({B_{1}}^{2}+{C_{1}}^{2}\right)}Y=aC_{1}\sin u \end{array}\right.$$

Substituting (7) into (6) yields the parametric equation of *Y* with respect to *u*. Subsequently, substituting the parametric equations of *X* and *Y* with respect to *u* back into (5) yields the parametric equation of *Z* with respect to u. The parametric equations of *X*, *Y*, and *Z* are shown in Equation (8), where 0 ≤ *u* ≤ 2π:

(8) $$\;\left\{\begin{array}{c} X=h\sqrt{\frac{\left({B_{1}}^{2}+{C_{1}}^{2}\right)}{\left({A_{1}}^{2}+{B_{1}}^{2}+{C_{1}}^{2}\right)}}\cos u\\ Y=\frac{h}{\sqrt{{B_{1}}^{2}+{C_{1}}^{2}}}\left(C_{1}\sin u-\frac{A_{1}B_{1}}{\sqrt{{A_{1}}^{2}+{B_{1}}^{2}+{C_{1}}^{2}}}\cos u\right)\\ Z=-h\left(\frac{B_{1}}{\sqrt{{B_{1}}^{2}+{C_{1}}^{2}}}\sin u+\frac{A_{1}C_{1}}{\sqrt{{A_{1}}^{2}+{B_{1}}^{2}+{C_{1}}^{2}}\sqrt{{B_{1}}^{2}+{C_{1}}^{2}}}\cos u\right) \end{array}\right.$$

Ultimately, the parametric equation for *O*_1_, as in Figure 1, can be obtained as follows:

(9) $$\;\left\{\begin{array}{c} x_{O_{1}}=x_{mid}+X\\ y_{O_{1}}=y_{mid}+Y\\ z_{O_{1}}=z_{mid}+Z \end{array}\right.$$

When both *B*_1_ and *C*_1_ are 0, Equation (9) will fail, and the spatial circumferential parametric equation is converted to the parametric equation of a circular line on the *x* = *x*_mid_ plane:

(10) $$\;\left\{\begin{array}{c} x_{o_{1}}=x_{mid}\\ y_{o_{1}}=y_{mid}+h\cos u\\ z_{o_{1}}=z_{mid}+h\sin u \end{array}\right.$$

The above parametric equation represents the trajectory of the outer circle center *O*_1_ rotating around the axis *PQ.*

Then, take the point *O*_1_ as the center of the circular line, *R* as the radius, and cross-multiply the vector ***PO***_1_ with the vector ***QO***_1_ to obtain the normal vector (*A*_2_, *B*_2_, *C*_2_) to construct the parametric equations of the spatial circumference of the arc, which is also divided into two cases, 0 ≤ *v* ≤ 2π, when both *B*_2_ and *C*_2_ are not 0:

(11) $$\left\{\begin{array}{c} x_{o}=x_{o_{1}}+R\sqrt{\frac{\left({B_{2}}^{2}+{C_{2}}^{2}\right)}{\left({A_{2}}^{2}+{B_{2}}^{2}+{C_{2}}^{2}\right)}}\cos v\\ y_{o}={y_{o}}_{1}+\frac{R}{\sqrt{{B_{2}}^{2}+{C_{2}}^{2}}}\left(C_{2}\sin v-\frac{A_{2}B_{2}}{\sqrt{{A_{2}}^{2}+{B_{2}}^{2}+{C_{2}}^{2}}}\cos v\right)\\ z_{o}={z_{o}}_{1}+-R\left(\frac{B_{2}}{\sqrt{{B_{2}}^{2}+{C_{2}}^{2}}}\sin v+\frac{A_{2}C_{2}}{\sqrt{{A_{2}}^{2}+{B_{2}}^{2}+{C_{2}}^{2}}\sqrt{{B_{2}}^{2}+{C_{2}}^{2}}}\cos v\right) \end{array}\right.$$

When both *B*_2_ and *C*_2_ are 0,

(12) $$\left\{\begin{array}{c} x_{o}=x_{o_{1}}\\ y_{o}=y_{o_{1}}+r\cos v\\ z_{o}=z_{o_{1}}+r\sin v \end{array}\right.$$

Equations (9) and (10), along with Equations (11) and (12), are the four forms of the parametric equations for TLS obtained according to different combinations of each other.

### 3.3. Solving the Intersection of Three Torus-like Surfaces

Most of the methods for intersecting two curves use tracking method, where the definition of the step size is difficult after the initial position of the intersection point is determined. In order to ensure accurate intersection, the traditional approach is to repeatedly experiment with the optimal step size to adapt to the current surface intersection. However, the sizes of torus-like surfaces change dynamically in application scenarios, so it is obvious that a fixed step size cannot be well adapted to torus-like surface intersection. Different from the traditional methods, this paper adopts a method to intersect torus-like surfaces with an adaptive change of step size according to the characteristics of the torus-like surfaces, which relates the step size to the radius of curvature at the end of the intersecting line, so that the step size can be changed in real time according to the real characteristics of the surfaces. The points on the surface intersection line are fitted with five non-uniform B-spline curves to obtain a higher accuracy of localization.

Four PCs allow the construction of parametric equations for three TLSs at three different locations.

The intersection between two of the parametric TLSs is transformed into a set of parametric curves and a parametric TLS, i.e., the problem is transformed into a problem of intersection between a parametric curve and a parametric surface. Let the two TLSs be P_1_ = P_1_(*u*_1_, *v*_1_), P_2_ = P_2_(*u*_2_, *v*_2_). Define a *u*-directed parametric curve on P_1_ as C(*u*), whose equation can be expressed as C(*u*) = P_1_(*u*_1_, $\bar{v_{1}}$), where the parameter $\bar{v_{1}}$ indicates that the parameter is fixed. *c*_0_ = P_1_(*u*_10_, $\bar{v_{1}}$) is a point on the parametric line C(*u*) of the torus-like surface P_1_, and *s*_0_ = P_2_(*u*_20_, *v*_20_) is a point on the torus-like surface P_2_. The vector distance between *c*_0_ and *s*_0_ is defined as *τ* = *c*_0_ − *s*_0_, and an accuracy threshold *ε* is set; when |*τ*| ≤ *ε*, it means that the distance between the two points is small enough to be used as the initial value of the intersection. The variable in the parametric line C(*u*) = P_1_(*u*_1_, $\bar{v_{1}}$) is u1, and the corresponding variables of surface P_2_ are *u*_2_ and *v*_2_, respectively. Let

(13) $$\left\{\begin{array}{l} \Delta u_{1}=u_{1}-u_{10}\\ \Delta u_{2}=u_{2}-u_{20}\\ \Delta v_{2}=v_{2}-v_{20} \end{array}\right.$$

A Taylor’s formula expansion of τ is obtained by neglecting the higher terms:

(14) $$\left\{\begin{array}{c} \tau \approx P_{1u}(u_{10},{\bar{v}}_{1})\Delta u_{1}-P_{2u}(u_{20},v_{20})\Delta u_{2}-\\ P_{2v}(u_{20},v_{20})\Delta v_{2} \end{array}\right.$$

Projecting the above equation into normed linear space and dot-multiplying P_1*u*_, P_2*u*_, and P_2*v*_ on each side of the equation yields three linear equations:

(15) $$\left\{\begin{array}{l} P_{1u}\cdot \tau =\left(P_{1u}\cdot P_{1u}\right)\Delta u_{1}-\left(P_{1u}\cdot P_{2u}\right)\Delta u_{2}-\left(P_{1u}\cdot P_{2v}\right)\Delta v_{2}\\ P_{2u}\cdot \tau =\left(P_{2u}\cdot P_{1u}\right)\Delta u_{1}-\left(P_{2u}\cdot P_{2u}\right)\Delta u_{2}-\left(P_{2u}\cdot P_{2v}\right)\Delta v_{2}\\ P_{2v}\cdot \tau =\left(P_{2v}\cdot P_{1u}\right)\Delta u_{1}-\left(P_{2v}\cdot P_{2u}\right)\Delta u_{2}-\left(P_{2v}\cdot P_{2v}\right)\Delta v_{2} \end{array}\right.$$

The above system of three equations in groups does not have a zero denominator or does not converge because the coefficient matrix is positively definite. Substituting *u*_10_ + ∆*u*_1_, *u*_20_ + ∆*u*_2_, *v*_20_ + ∆*v*_2_ into *τ* by using Taylor’s formula expansion for the point *c*_0_ and iterating based on the normed linear space projection method [25] until |*τ*| ≤ *ε*, the initial value of the intersection point is obtained, and whether the intersection point is on the third TLS or not is determined, and if it is, it is saved as a row of intersection candidates.

The next intersection point is solved next, and so on; a series of intersections can be obtained to acquire the whole intersection line. Requirements to take the next intersection are necessary to analyze the point in the space curve of the characteristic parameters. *c*_0_ = *c*(*s*_0_) is the curve *c* on a point, *s*_0_ is the arc length parameter, and then *c*_0_ neighboring points can have a Taylor expansion expressed as follows:

(16) $$c(s_{0}+\Delta s)=\dot{c}(s_{0})+\dot{c}(s_{0})\Delta s+\frac{1}{2^{\prime }}\ddot{c}(s_{0})\Delta s^{2}+o(\Delta s^{2})$$

There are in differential geometry:

(17) $$\dot{c}(s_{0})=\alpha ;\ddot{c}(s_{0})=k\beta$$

In the [*c*_0_, *α*_0_, *β*_0_] coordinate system, defining the point at *c*_0_ as the starting point, there is an arc length parameter of *s*_0_ = 0, step size of Δ*s* = *s*, and the neighboring points can be expressed as follows:

(18) $$c(s)=c_{0}+\alpha_{0}s+\frac{1}{2}ks^{2}\beta_{0}$$

where *α*, *β*, and *k* are the tangent vectors, principal normal vectors, and curvature of the space curve *c* at the point *c*_0_. Based on *α* and *β*, the close planes can be obtained, which in turn gives the geometric structure of the point *c*_0_ on the space curve [26]; we can solve for *α*, *β*, and *k*, respectively:

(19) $$\begin{array}{l} \cos\theta =n_{1}\cdot n_{2}\\ \dot{u}=\frac{du}{ds};\dot{v}=\frac{dv}{ds}\\ k_{n}=\frac{II}{I}=L{\left(\dot{u}\right)}^{2}+2M\dot{v}+N{\left(\dot{v}\right)}^{2}\\ \alpha =\frac{n_{1}\times n_{2}}{\left|n_{1}\times n_{2}\right|}\\ k=\left|k\right|=\frac{\sqrt{{\left(k_{n}^{1}\right)}^{2}+{\left(k_{n}^{2}\right)}^{2}-2k_{n}^{1}k_{n}^{2}\cos\theta }}{\left|\sin\theta \right|}\\ \beta =k/k \end{array}$$

where ***n***_1_ and ***n***_2_ are the normal vectors at the point *c*_0_; *k*_n_ is the normal curvature of each surface at that point obtained from the first and second fundamental forms of the surface; *L*, *M*, and *N* are the coefficients in the second fundamental form; and ***k*** is the curvature vector.

The intersection, which is solved by plugging *α*, *β*, and *k* combined with *s* into Equation (18), is an approximation, and at this time, the step size *s* is unknown, and if it is set artificially based on experience, the result is not stable and efficient enough, and we found that TLS intersection lines have more obvious curvature changes in different regions. So, we correlate the step-size *s* with the radius of curvature at the end of the intersection line according to [27] so that the step size can be changed in real time based on the real characteristics of the surface; points on the intersection line are fitted using fivefold non-uniform B-spline curve.

(20) $$\left\{\begin{array}{l} k_{r}=\left|C^{\prime \prime }(u)\right|/{\left(1+C^{\prime }{\left(u\right)}^{2}\right)}^{3/2}\\ r=1/k_{r}\\ s=2\times \sin(\theta /2)\times r;\theta \leq 2\times \arccos(1-e\times k_{r}) \end{array}\right.$$

where C(*u*) is the fivefold non-uniform B-spline curve equation, *u* = 1 when the endpoint of C(*u*), *r* is the radius of curvature on C(*u*), and *k_r_* is the curvature of C(*u*). When *k_r_* is less than 0, it indicates that the fitted curve curves are bent clockwise along the direction of advancement, and when it is greater than 0, it indicates that it is bent counterclockwise. *e* denotes the threshold of accuracy, which is generally taken to be 0.001 mm. The points obtained at this time are not the exact solution, and it is also necessary to iterate to obtain a more accurate value [28].

In summary, two points closer to the point *c*(*s*), denoted as *p*_1_ and *p*_2_, are firstly selected on the two TLSs P1 and P2, respectively, with *p*_1_ belonging to P1 and *p*_2_ belonging to P2.

If there is a point *p*_1_ and *p*_2_ converging to the neighboring point *c*(*s*), the point should meet the set accuracy |*p*_1_ − *p*_2_| ≤ *ε*. If *p*_1_ and *p*_2_ cannot converge at the neighboring point, *p*_2_ is defined as an approximation of the intersection point. Then, the above computation process is repeated continuously.

If the condition is satisfied, its coordinates need to be substituted into the equation of the third TLS in order to determine whether the point is on this TLS or not. If the distance *d* from the midpoint of the axis to the intersection point satisfies the following condition, then *γ* is the angle between the vector from the midpoint of the axis to the intersection and the axis vector.

(21) $$\tau \geq \left\{\begin{array}{l} \left|d-(h+R)\right|;\gamma \approx \frac{\pi }{2}\\ \left|d-(\sqrt{R^{2}-h^{2}}/\cos\gamma )\right|;0<\gamma <\frac{\pi }{2} \end{array}\right.$$

The point is added to the candidate set of intersections. Then, the exact point *c*(*s*) found by iteration is used as the initial intersection, and the location of the next intersection continues to be counted until the boundary of the surface is traced.

**Figure 3:**
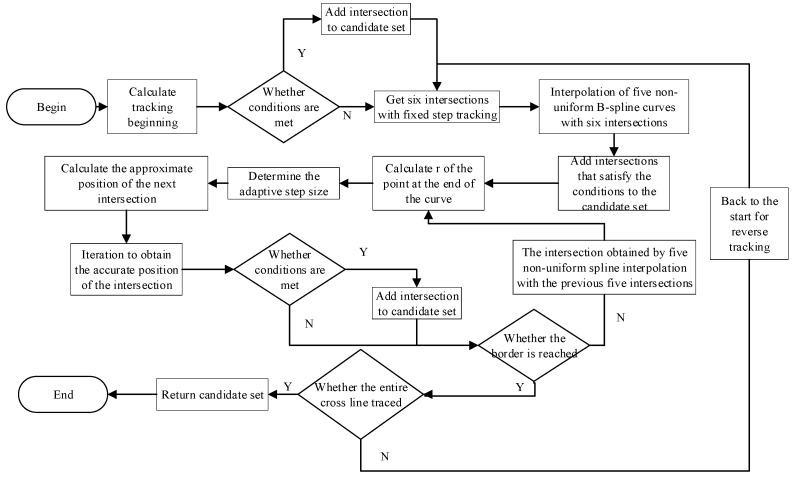
Process for obtaining the candidate set of intersections.

The approximate process for solving the intersection is as Figure 3:

The three TLS equations are constructed in such a way as to obtain the circumferential angles *ξ*_1_, *ξ*_2_, and *ξ*_3_, respectively. The angle of circumference *ξ*_1′_, *ξ*_2′_, and *ξ*_3′_ formed by the resulting point of intersection and the two endpoints on the axis, if it satisfies *ξ*_1′_ > *ξ*_1_ or *ξ*_2′_ > *ξ*_2_ or *ξ*_3′_ > *ξ*_3_, shows that the intersection is on the minor arc opposite the axis and should be excluded.

There may be more than one candidate for the intersection, which needs to be filtered according to the normal vectors of the three TLSs’ tangent at the intersection. If the intersection meets the requirements, then it must meet the three TLSs of the visual cone on this intersection overlap, i.e., the upper vector of the three must be at the intersection of the same tangent. Thus, it can be based on the intersection of the tangent surface of the normal vector to determine whether it is colinear or not. If not, then rule out the intersection. In practice, there will still be a very small error, so it is necessary to set a threshold, the normal vector between the two cross-multiplications of the modulus, to do the error evaluation and set it to 1 × 10^−11^ for the best results after many tests. After filtering, there may still be more than one intersection, so direction vectors should be utilized for further filtering.

### 3.4. Solving Forward Vector and Upper Vector

The world coordinates of the intersection *O* are shown in Section 3.3, and we can easily obtain the distances of *OP* and *OQ*. Find the world coordinates *S*_0_ and *S*_1_ of *P*, *Q* projected on the imaging plane by the proportionality of *OP* and *OQ* to *Op* and *Oq* derived in Section 3.1:

(22) $$\begin{array}{l} S_{0}(x,y,z)=(O(x,y,z)-P(x,y,z))\cdot (\frac{Op}{OP})+O(x,y,z)\\ S_{1}(x,y,z)=(O(x,y,z)-Q(x,y,z))\cdot (\frac{Oq}{OQ})+O(x,y,z) \end{array}$$

Follow the above equation to find the world coordinates of third point projected on the imaging plane, assumed to be *S*_2_.

The forward vector ***f*** is obtained by vector cross-product:

(23) $$f=S_{0}S_{1}\times S_{1}S_{2}$$

Since these four feature points are ordered beforehand on the image coordinate system according to their positions from top to bottom and left to right, it can be guaranteed that the forward vector is the orientation of the view cone. At the same time, it can be guaranteed that the upper vector of the subsequent solution always points to the camera directly above. If there are still multiple intersection solutions, use whether the angle between ***OP*** and ***f*** is greater than half of the diagonal viewing angle of the view cone. If it is greater, it means the point is a mirror solution; exclude this type of intersection and leave the intersection with the smallest error as the ultimate solution.

**Figure 4:**
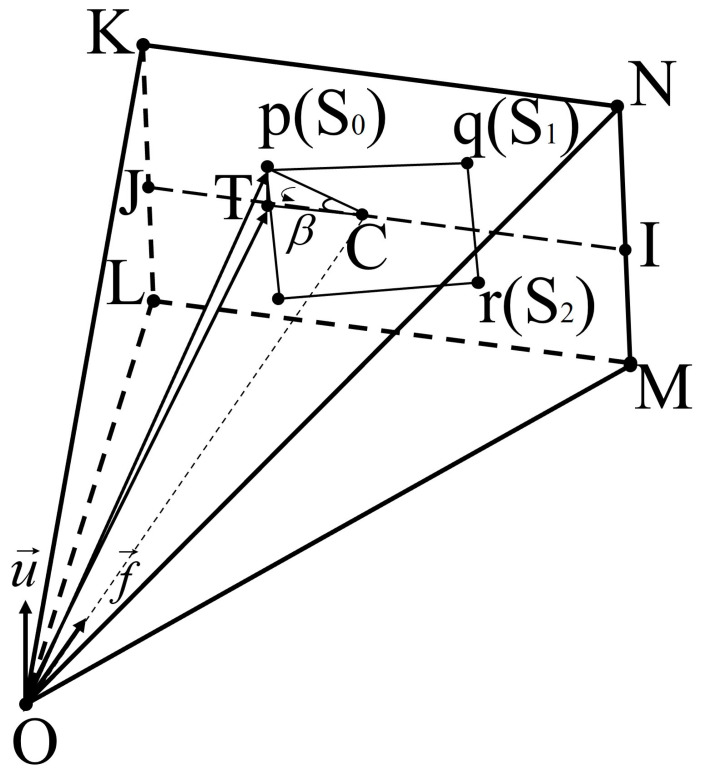
Solving the upper vector based on the forward vector.

To solve the upper vector based on the front vector, it is also necessary to find a point *T* on the centerline *JI* on the imaging plane and parallel to the *KN* side that is different from the pixel position of point *C*. As shown in Figure 4, select the image coordinate point *p* and determine the image coordinates of point *T*, whose horizontal coordinate is the abscissa of *p*, and the vertical coordinate is the ordinate of *C*. In this respect, a right triangle *pTC* can be constructed, and *β* is found based on the Pythagorean theorem, and then the vector ***OT*** can be obtained from the vector ***OS***_0_ rotated around the forward vector ***f*** by an angle *β*. The upper vector ***u*** of the camera position point *O* can be obtained by cross-multiplying the unit vector of ***OT*** with the forward vector ***f***.

The TLS solution yields the forward and upward vectors of the camera in the world coordinate system, which can be extrapolated by Rodriguez’s formula to obtain the rotation matrix ***R***. Combined with the intersection coordinate *O*, the translation vector ***t*** is easily obtained.

## 4. Experiments and Analysis of Results

Given the true query camera center *O*_g_ = −***R***_g_^T^***t***_g_ and the estimated query camera center *O*, the position error is calculated as ||*O* − *O*_g_||. The rotation error is calculated as ||ln (***R***_g_***R***^T^)||. While arccos((trace(***R***) − 1)/2) is often used as the rotation error metric, using ||ln (***R***_g_***R***^T^)|| is more stable for small angle rotations.

To support the UP2P algorithm application, the method in [29] is utilized to decompose the ground truth rotation matrix ***R***_g_ into ***R***_Y_ rotated around the *Y*-axis and ***R***_XZ_ rotated around the X-Z plane vectors, i.e., ***R***_g_ = ***R***_Y_***R***_XZ_. ***R***_XZ_ is taken as the input, and the only rotation unknown *θ* is the rotation around the *Y*-axis.

To support the IPPE algorithm application, three additional PCs are generated by sampling 2D points around point *x* in the reference image using the method in the P1AC literature. Then, 3D points are obtained by intersecting a ray with the plane where the normal vector ***n*** is located through the camera as a starting point. Additionally, 2D points are found by applying an affine transformation to simulate four 3D points coplanar; three of these four points are not co-linear with each other and will be used as inputs to the method based on four PCs.

### 4.1. Synthetic Data Experiments

We use a data generation method adapted from [30], assuming that reference and query cameras with the same intrinsic are randomly placed at a distance of [1,2] around the origin and that both cameras are oriented towards a random target point in the range of [−0.5,0.5]^3^. To establish a single-point correspondence between the two cameras, we randomly select a 3D point from N (0, I_3×3_), then take a random plane normal vector at that point and project it onto the imaging surface of the two cameras using a focal length of 400. Then, the affine transformation between two cameras is computed from the local homography matrix using the method of reference [31] by transforming the two cameras and the 3D point so that the reference camera’s external reference matrix with respect to the origin is [**I**|**0**]. The translation vectors and target 3D points in the synthesized data are generated by the rand() function of C++, and these random numbers obey a normal distribution; the rotation matrix between the reference and query cameras is transformed by a random unit quaternion that follows a uniform distribution law on SO(3).

#### 4.1.1. Numerical Stability

**Figure 5:**
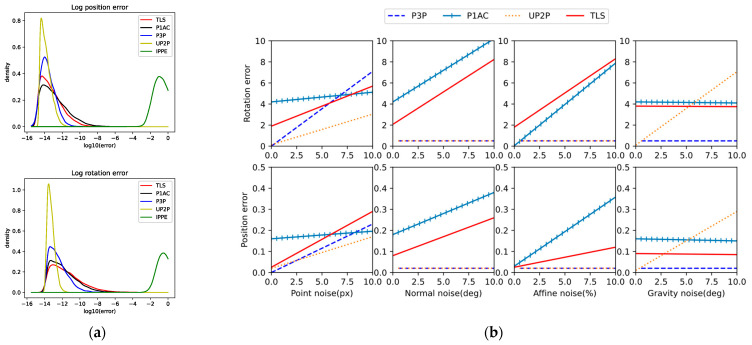
Results of synthetic data experiments. (**a**) Analysis of numerical stability with zero noise added to observations. The plots are estimates of the distribution produced by Gaussian kernel-density estimation. Top: Log rotation error. Bottom: Log position error. (**b**) The median error of two-dimensional observations, normal vectors, affine transformations, and gravity vectors relative to noise. The *x*-axis shows the std. dev. of the noise added.

We tested the numerical stability of each solver on 10,000 random problem instances with no noise added to the observations. Figure 5a shows the density plots of log rotation and position errors for each solver. Except for IPPE, which performs poorly due to the unavoidable errors in the three virtual PCs generated by the P1AC literature’s method. IPPE is quite sensitive to PCs, whether it is completely coplanar. On the contrary, the TLS method is not much affected. It is known that TLS adapts itself according to the point positions and solves stably even at the four points of the near coplanar plane without any peaks higher than 10^−2^. The fact that more than 99.9% of the points in P1AC and TLS have an error of less than 10^−5^ suggests that these solvers are able to produce usable results.

#### 4.1.2. Noise

We test the sensitivity of various methods by adding multiple types of noise to the generated data.

- Point noise: add Gaussian noise to 2D observations;
- Normal noise: randomly select angles in the Gaussian distribution, and the normal vector is rotated according to the angles;
- Affine noise: adds Gaussian noise to the elements of the 2 × 2 affine transform, and the level of Affine noise is chosen according to the selected percentage error, e.g., with a percentage of r, the noise added to a_ij_ is r∙a_ij_;
- Gravity noise: perform a random rotation of ***R***_XZ_, with the rotation angle taken from a normal distribution.

The TLS, P1AC, UP2P, and P3P solvers were tested in a range of noise settings for each noise type, and the median error was calculated for 10,000 iterations at each setting. For the noise types, which varied in each setting, the default noise settings were 1-pixel noise, 1° normal vector noise, 4% affine noise, and 0.5° gravity-oriented noise.

From the results in Figure 5b, it can be seen that since the solution process of P1AC relies heavily on normal vectors and affine matrices, it is extremely sensitive to normal vector noise and affine noise, but since the algorithm only uses a single point correspondence, it is less affected by point correspondence; UP2P introduces gravity vectors as an input, which is not only affected by point noise, but also quite sensitive to gravity vector noise; P3P does not P3P does not utilize normal vectors and affine matrices in the solution process, nor does it involve gravity vectors, but it is more affected by the feature points; similarly, the model of TLS is constructed based on geometric constraints between 3D points and their projected points, and thus it is largely affected by the point correspondences.

Theoretically, TLS is not affected by normal and affine noise. In order to compare with P1AC in normal noise and affine noise experiments, the three virtual points inputted into TLS are generated according to the method in P1AC. This process utilizes the normal vector and affine matrix, which is equivalent to indirectly affecting the point correspondence through normal noise and affine noise. The results show that as the normal noise and affine noise increase, the magnitude of error change is less affected by these noises than P1AC, i.e., the TLS can solve the high-accuracy solution more stably.

The accuracy of TLS is weaker than P1AC or even P3P with the addition of roughly 5px point noise to the default observations, which is not too much of a concern in practice, where the RANSAC algorithm is generally used to exclude feature pairs with high point noise during feature matching, and will be previewed in the subsequent real data experiments. Subsequent real data experiments will pre-process the matched pairs using RANSAC.

#### 4.1.3. Time

We compared the average time required by each solver to solve a single configuration problem out of 10,000 random problems. All solvers were implemented in C++, computed using an Intel(R) Core(TM) i7-7700 (Intel Products (Chengdu) Co., Ltd., Chengdu, China), and used the UP2P and P3P solvers from the PoseLib library, with the execution of IPPE provided by the solvePnP function in OpenCV and P1AC using the author’s official release. The results are shown in Table 1. With the exception of IPPE, the average time for the rest of the solvers is less than 2.8 μs, and solving is quite fast.

#### 4.1.4. Robust Estimation

We investigated the performance of TLS and P3P in robust estimation with an increasing proportion of outliers.

To analyze the trade-off between having a small minimum sample size and a large noise sensitivity, we tested a range of outlier ratios from 0 to 0.9, with 10,000 trials at each setting, with the default settings for noise consistent with those in the noise experiments. In each trial, we randomly generated 1000 PCs, replacing some of the correspondences with random values based on the desired outlier ratio. We then run vanilla RANSAC or LO-RANSAC [32] using TLS or P3P as the minimum solver in local optimization. LO-RANSAC reduces the effect of noise on the minimum solver by combining iterations with local optimization (LO) to increase the set of interior points from the initial estimate. The effect of noise on the minimum solver is minimized.

The average error of each method is stable over a range of outlier ratios. Table 2 shows the average error for both methods for all outlier ratios. When vanilla RANSAC is used, TLS is less accurate than P3P at this noise level, and when LO-RANSAC is used, although TLS has a slightly higher error than P3P, both methods have very low error ratios, proving their effectiveness. Note that on real data, TLS performs substantially better than P3P.

**Figure 6:**
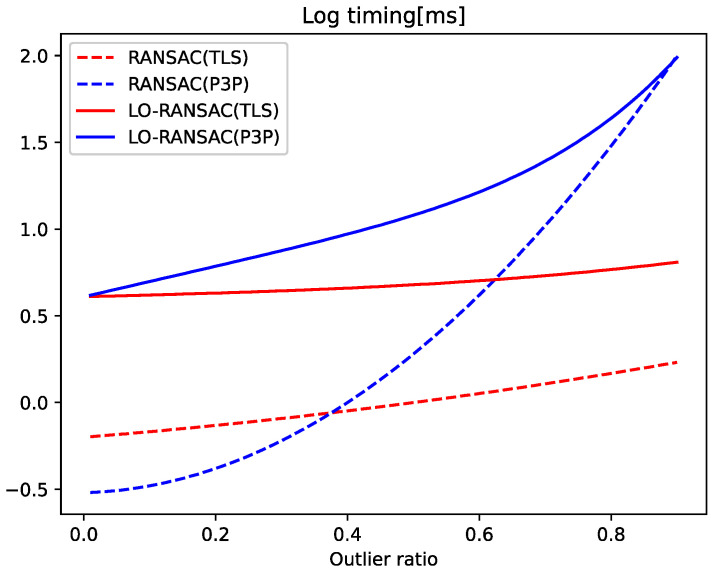
Average timing for each robust estimation method across a range of outlier ratios.

Figure 6 shows the average time for each method. With vanilla RANSAC, TLS is faster than P3P at outlier ratios of about 0.37; with LO-RANSAC, TLS is faster than P3P at all outlier ratios. This result shows that our TLS solver is accurate enough to provide a good initialization for LO-RANSAC and reaches convergence faster than P3P, even at lower outlier ratios. In the real data experiments (Section 4.2), in order to be consistent with the state-of-the-art, we used Graph Cut RANSAC (GC-RANSAC) [6], which improves on LO-RANSAC by adding graph cut optimization to the LO iterations. We did not use GC-RANSAC in the synthetic data experiments because the observations are randomly generated and not spatially coherent.

Mirror solutions, i.e., pairs of solutions that are rotated by exactly 180 degrees from each other, may occur in the TLS solution. Although these solutions will be filtered out by Ransac, we have added an extra restriction to the algorithm, i.e., if the angle between a ray vector emitted from the position out and the angle formed by the forward vectors is obtuse, the solution can be recognized as a mirror solution, and another solution is taken.

### 4.2. Real Data Experiments

To compare the performance of the methods on large-scale image localization datasets, we use the Cambridge Landmarks [33] and Aachen Day-Night v1.1 [34] datasets. These two datasets are more commonly used in the image-based visual localization literature.

The Cambridge Landmarks dataset consists of six scenes from Cambridge, UK, each recorded from multiple video sequences taken on a smartphone, capturing different parts of the city. From the sequences recorded for each scene, one part represents the database image for that scene, and the other part is used to acquire query images. The ground truth and internal intrinsic parameter calibrations of all images were determined using Visual SFM [35] Visual (SfM) software (v0.5.26). The reference and query images are taken from the same walking paths under the same lighting and similar environmental conditions, which increases the difficulty of localization due to the fact that the angles and shooting distances are not sampled from similar trajectories. Localization performance was generally determined by comparing the median errors of position and orientation. In addition, we evaluated the proportion of images (i.e., recall) for which the positional estimates were within 5 cm/1°, 10 cm/1°, and 20 cm/1° of the actual position.

The Cambridge Landmarks dataset was taken under the same lighting and similar environmental conditions. We choose the dataset Aachen Day-Night v1.1 to compare the localization performance of each algorithm based on a daytime reference image for the daytime query Day and a nighttime query Night, which implies that there is no nighttime image in the reference image. These images were taken at different times and years over a two-year period. Therefore, these images cover a wide range of lighting and weather conditions, as well as temporary occlusion of construction sites and building changes that are not present in the 3D model. The ground truth of the daytime reference images was established via COLMAP [36], and the nighttime query images were re-referenced to the initial pose for alignment [34]. We followed a common evaluation protocol to report the percentage of images localized within three error thresholds (0.25 m/2°, 0.5 m/5°, 5.0 m/10°).

There are various ways to obtain affine features from real images. Experiments will use the following two feature extraction and matching algorithm approaches to obtain valid 2D feature point pairs as well as affine matrices. One is to use a local feature detector (e.g., DoG [37]) to estimate the location and scale of key points and then use patch-based AffNet [38] to obtain the affine shapes. Finally, a patch-based descriptor such as HardNet [39] is applied. The other uses SuperPoint [40] for feature detection, LightGlue [41] for feature matching, and finally SelfScaleOri [42] to obtain the image feature orientation and scale. Based on the orientation and scale, the affine matrix *A_i_* of the *i*-th feature is approximated as *S_q__i_R_β__i_*, where *S_q__i_* is a diagonal matrix scaled by the detected scale factor *q_i_* scales uniformly along the axis, and *R_β__i_* belonging to SO(2) is rotated by the estimated angle *β_i_* ∈ [0, 2π). The normal vector is estimated using the 200 nearest neighbors of each point in the SfM point cloud. The direction of gravity is extracted from the ground truth of the rotation matrix as UP2P input.

In these experiments, our aim is to analyze the usefulness of TLS in practice. For this purpose, we use GC-RANSAC and fine-tune each solver so that they can achieve the best performance possible.

As can be seen from Table 3 and Table 4, the P1AC method has a smaller average error and a higher percentage of recall in almost all scenarios. We also find that TLS has comparable results with P1AC in terms of positional error, but the difference is larger in terms of rotational error. According to the TLS principle, the higher the positional solving accuracy, the smaller the rotational error will be. Next, we attempt to utilize SuperPoint + LightGlue + SelfScaleOri (SP + LG + SSO) to obtain the image feature matching, feature orientation, and scale to test performance.

As shown in Table 5, all the solvers have improved greatly in extracting feature information using SP + LG + SSO, especially the TLS method in this paper, which has more significant improvement in position and rotation due to more and more accurate 2D-3D valid matches obtained. It is worth noting that the rotation error of P1AC is instead larger than before. This is most likely because the derived affine relation approximation from the orientation and scale obtained by SSO is more biased than the one obtained by AffiNet.

Aachen Day-Night is a feature extracted using both DoG + AffiNet + HardNet (DG + AN + HN) and SP + LG + SSO. Since we do not know the direction of gravity, it is assumed to point directly downward (i.e., [0, −1, 0]^T^). This is usually a safe assumption since people tend to take photos upright. The results are shown in Table 6 and Table 7.

Observation of Table 6 shows that most of the solvers achieve good results in the Day dataset but have uneven results at Night, indicating that DoG is not stable and efficient enough to capture valid 2D-3D matches at night.

Since the application of SP + LG + SSO in the original Day dataset enabled the various methods to achieve close to 100% recall, we resized the images so that their longest dimension is 800 pixels at maximum. On the Day sequence, all algorithms had reasonably good precision, except for P3P, which was slightly weaker. Overall, TLS performs best. Although P1AC achieves high recall on 5 m/10°, it is not accurate enough at tighter thresholds. In the Night sequence, TLS achieves higher accuracy with SP + LG + SSO feature extraction.

We used DG + AN + HN for feature extraction and GC-RANSAC for filtering to report the average time of various methods. As we expected, P3P has the shortest time in the Cambridge Landmarks dataset, P1AC has the shortest time for the Day sequences in Aachen Day-Night, and IPPE is faster for the Night sequences. TLS does not have much of an advantage in terms of time due to the fact that it takes a longer time to find intersections on surfaces by step tracking. However, this approach has many beneficial properties, such as robustness and determinism. This has been demonstrated in various data experiments and is even faster in some cases.

For completeness, we also recorded the time required for feature extraction, with DoG + HardNet taking 0.21 ms per feature and DoG + HardNet + AffNet taking 0.64 ms. The results are shown in Table 8.

### 4.3. Navigation Application Effect

Using the motion tracking technology that fuses the camera and IMU data as the basis, the A* algorithm does the navigation path planning to realize the AR indoor navigation system application. The application is deployed to Redmi K50 cell phones to test the application effect in a design exhibition hall.

**Figure 7:**
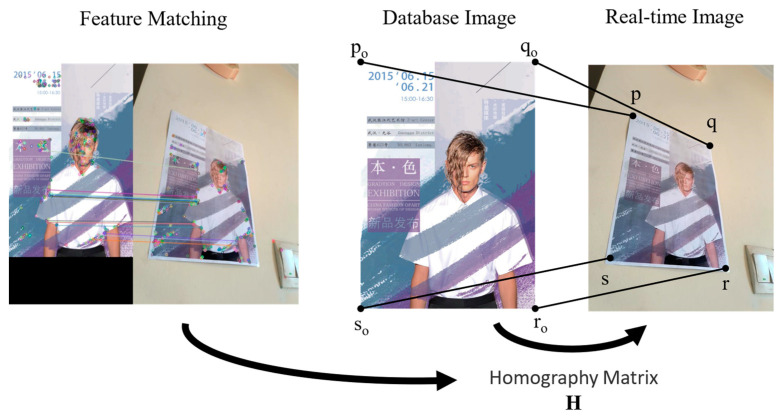
The process of feature matching to obtain four PCs.

Figure 7 shows the process of feature matching to obtain four PCs. First, we mark and measure the key points that need to be navigated in the scene, consider the posters in the scene as markers, make the four corners of the poster as reference points, record their positions, and store this information in the database.

**Figure 8:**
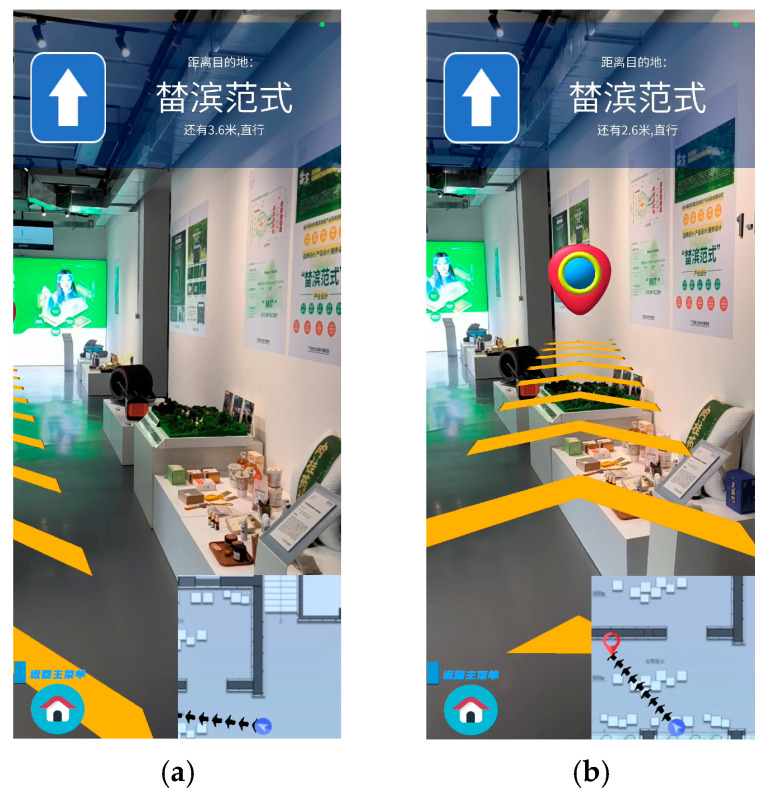
The path navigation performance of the AR indoor navigation system. (**a**) Without TLS visual localization; (**b**) with TLS visual localization.

By using the feature point pairs obtained from Harris corner detection and ORB operator matching, we were able to obtain the homograph ***H*** between the poster in the database and in the application scene. Through ***H***, we were able to determine four PCs of the poster corners. Combined with the TLS visual localization, we corrected the camera’s position and thus adjusted the pointing of the navigation route. The correction effect is shown in Figure 8.

Figure 8 shows the path navigation effect of the AR indoor navigation system. Figure 8a represents the navigation effect obtained only by relying on the motion tracking technology, which does not allow the arrow path to accurately guide to the “榃滨范式” destination due to the cumulative error when moving; Figure 8b represents the effect of real-time correction of the device’s position by combining with the TLS visual localization in the system, which enables the device to accurately perceive its own positioning in the scene, thus forming a more accurate navigation route to the destination, reflecting the accuracy of TLS.

**Figure 9:**
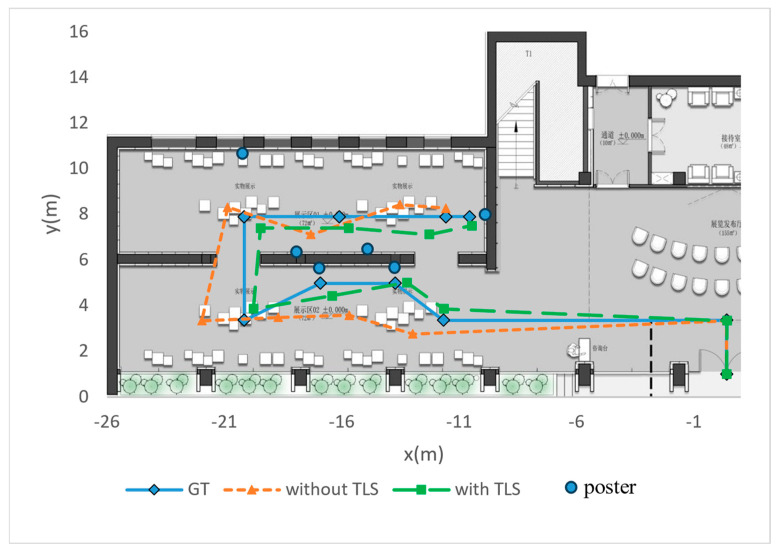
Aerial view of the navigation path.

We also record ten sets of key localization points and connect them into paths to compare the accuracy of TLS. As shown in Figure 9, the background image is an aerial view cross-section of the pavilion, the paths consisting of diamond-shaped points are ground truth (GT) measured in the scene, and the paths consisting of triangular and square points are visual localization with/without TLS, respectively.

As can be seen from the aerial view, relying only on the motion tracking of the mobile device will make the localization have a great deal of randomness, thus seriously affecting the quality of the navigation. After applying the visual localization algorithm in this paper, the obtained navigation paths can better fit the pre-set navigation routes, reflecting the practicality of TLS.

The superposition of AR navigation paths and video images is another segmentation theme of the navigation guide project, which uses the A* algorithm to plan a feasible path based on the camera position and the target position and real-time visualization of the path and the target point in Unity, which is discussed in a separate contribution by the author due to the long length of the algorithm.

## 5. Conclusions

In this paper, the camera’s absolute position is used as the entry point. Three torus-like surface geometric constraints are constructed by combining the four PCs with the camera intrinsics and focal length. The problem of solving the position of four PCs is transformed into the surface intersection. We utilize the step-size adaptive tracking algorithm to quickly find the available intersections and then solve the rotation matrix. Experiments show that our method has excellent performance in terms of stability, time, and accuracy. Its accuracy and usefulness in navigation application testing are demonstrated. Future work will be centered around constructing three torus-like surfaces with fewer PCs. We will also explore what geometric constraints to apply in this situation to obtain the optimal positional solution.

## Figures and Tables

**Table 1:** Average timing in μs over 10,000 trials.

	P3P	UP2P	IPPE	P1AC	TLS
Time (μs)	0.54	0.33	25.27	2.73	2.37

**Table 2:** Average rotation and pose error for each robust estimation method on synthetic data over a range of outlier ratios.

Robust Estimation	TLS		P3P	
	Rot (°)	Pos.	Rot (°)	Pos.
RANSAC	0.462	0.010	0.057	0.002
LO-RANSAC	0.079	0.002	0.017	0.001

**Table 3:** Cambridge Landmarks median position (centimeters) and rotation (degrees) errors of GC-RANSAC combined with various solvers when using DoG + AffiNet + HardNet.

	Position (cm) ↓					Rotation (°) ↓				
Scene	P3P	P1AC	UP2P	IPPE	TLS	P3P	P1AC	UP2P	IPPE	TLS
Great Court	8	**6**	**6**	7	**6**	0.1	0.09	**0.08**	0.1	0.12
King’s Col.	11	**7**	8	10	8	0.16	**0.14**	0.15	0.21	0.20
Old Hospital	9	7	7	8	**5**	0.14	**0.12**	0.13	0.13	0.15
Shop Façade	5	**2**	3	4	3	0.13	0.13	0.14	**0.12**	0.14
St Mary’s Ch.	7	**5**	**5**	6	6	0.2	**0.17**	**0.17**	0.19	0.19
Street	30	22	43	221	**20**	0.58	0.36	0.79	**0.26**	0.42
Avg.	11	8	12	43	**8**	0.22	**0.17**	0.24	**0.17**	0.24
Weighted avg.	22	**14**	32	152	15	0.41	0.26	0.52	**0.21**	0.36

**Table 4:** Cambridge Landmarks recalls (in percentages), at 0.1 m/1° and 0.2 m/1° of GC-RANSAC combined with various solvers when using DoG + AffiNet + HardNet.

	Recall (0.1 m/1°) ↑					Recall (0.2 m/1°) ↑				
Scene	P3P	P1AC	UP2P	IPPE	TLS	P3P	P1AC	UP2P	IPPE	TLS
Great Court	61.7	73.5	73.8	65.3	**73.9**	86.4	**91.1**	90.1	86.2	90.3
King’s Col.	49.3	64.6	65.1	57.7	**65.8**	79.2	**86.9**	84.7	85.4	84.9
Old Hospital	58.7	**72.3**	68.4	62.5	69.1	79.8	**93.5**	89.3	85.3	89.5
Shop Façade	85.3	**87.6**	86.3	84.3	85.8	94.4	**95.1**	93.1	92.3	92.7
St Mary’s Ch.	61.6	**72.5**	70.1	67.5	72.1	80.7	**87.6**	85.2	84.3	85.7
Street	10.6	**19.7**	16.5	19.1	17.2	17.8	**29.6**	23.9	26.7	24
Avg.	54.5	**65.03**	63.37	59.4	63.98	73.05	**80.63**	77.72	76.7	77.85
Weighted avg.	30.45	**40.61**	38.21	37.39	38.54	43.89	**53.52**	49.34	50.1	49.69

**Table 5:** Cambridge Landmarks average median position (centimeters) and rotation (degrees) errors and average recalls (in percentages) at 0.1 m/1° and 0.2 m/1° of GC-RANSAC combined with various solvers when using SP + LG + SSO.

Methods	Position (cm)/Rotation (°) ↓	Recall (0.1 m/1°)/Recall (0.2 m/1°) ↑
P3P	8/0.16	72.9/76.1
P1AC	7/0.23	85.7/88.6
UP2P	7/0.19	86.4/90.5
IPPE	13/**0.12**	84.2/88.4
TLS	**6**/0.13	**87.3**/**90.8**

**Table 6:** Aachen Day-Night pose error recalls, in percentages, at 0.25 m/2°, 0.5 m/5°, and 5.0 m/10° of GC-RANSAC combined with various solvers using DG + AN + HN.

	**Recall (0.25 m/2°) ↑**				
	P3P	P1AC	UP2P	IPPE	TLS
Day	61.9	62.2	62.1	62.0	**62.3**
Night	47.3	**51.4**	48.1	46.2	47.9
	**Recall (0.5 m/5°) ↑**				
Day	83.4	**84.6**	83.8	83.5	**84.6**
Night	60.3	**65.9**	64.7	63.8	62.7
	**Recall(5 m/10°) ↑**				
Day	96.0	95.9	95.7	96.0	**96.1**
Night	74.2	**82.3**	79.6	76.4	80.1

**Table 7:** Aachen Day-Night pose error recalls, in percentages, at 0.25 m/2◦, 0.5 m/5◦, and 5.0 m/10◦ of GC-RANSAC combined with various solvers using SP + LG + SSO.

	**Recall (0.25 m/2°) ↑**				
	P3P	P1AC	UP2P	IPPE	TLS
Day	63.4	64.3	65.8	66.1	**66.4**
Night	61.3	69.7	67.5	65.8	**70.1**
	**Recall (0.5 m/5°) ↑**				
Day	79.6	83.3	86.7	87.1	**87.9**
Night	77.5	**87.2**	86.4	86.6	87.1
	**Recall(5 m/10°) ↑**				
Day	92.4	**97.3**	96.8	97.1	**97.3**
Night	92.8	98.9	97.9	98.3	**99.0**

**Table 8:** Average time of GC-RANSAC using DG + AN + HN on Cambridge Landmarks and Aachen Day-Night datasets.

Scene	Time (s) ↓				
	P3P	P1AC	UP2P	IPPE	TLS
Great Court	**0.14**	0.26	0.17	0.21	0.28
King’s Col.	**0.19**	0.48	0.18	0.37	0.47
Old Hospital	**0.12**	0.41	0.41	0.38	0.45
Shop Façade	**0.13**	0.38	0.21	0.36	0.40
St Mary’s Ch.	**0.12**	0.38	0.72	0.29	0.37
Street	**0.11**	0.29	0.22	0.27	0.31
Day	0.23	**0.11**	0.14	0.18	0.13
Night	0.13	0.10	0.11	**0.06**	0.11

## Data Availability

Data are contained within the article.
